# Free triiodothyronine to free thyroxine ratio as a marker of poor prognosis in euthyroid patients with acute coronary syndrome and diabetes after percutaneous coronary intervention

**DOI:** 10.3389/fendo.2024.1322969

**Published:** 2024-04-08

**Authors:** Shen Wang, Yue Wang, Shuaifeng Sun, Fadong Li, Wenxin Zhao, Xinjian Li, Maomao Ye, Yufei Niu, Xiaofan Wu

**Affiliations:** Department of Cardiology, Beijing Anzhen Hospital, Capital Medical University, Beijing, China

**Keywords:** FT3/FT4 ratio, prognosis, acute coronary syndrome, percutaneous coronary intervention, diabetes

## Abstract

**Objectives:**

In recent years, the free triiodothyronine/free thyroxine (FT3/FT4) ratio, a new comprehensive index for evaluating thyroid function, which could reflect thyroid function more stably and truly than serum thyroid hormone level, has been demonstrated to correlate with the risks of diabetes and cardiovascular disease in euthyroid adults. However, the correlation between thyroid hormone sensitivity and long-term prognosis in euthyroid patients with acute coronary syndrome (ACS) and diabetes after percutaneous coronary intervention (PCI) remains unclear.

**Methods:**

A total of 1,786 euthyroid patients with ACS who successfully underwent PCI at Beijing Anzhen Hospital from August 2021 to April 2022 were included in our study, which was divided into three groups according to tertiles of thyroid hormone sensitivity index. Cox regression, Kaplan–Meier, and receiver operating characteristic analyses were applied to analyze the associations between the FT3/FT4 ratio with ACS and diabetes after PCI.

**Results:**

Our analysis indicated that a lower level of FT3/FT4 ratio in euthyroid patients with acute coronary syndrome (ACS) and diabetes after PCI showed significantly higher incidences of major adverse cardiac and cerebrovascular events (MACCE) when compared with a higher level of FT3/FT4 ratio. After adjusting for other covariates, patients with a lower level of FT3/FT4 ratio were negatively associated with the risk of MACCE than those with a higher level of FT3/FT4 ratio (adjusted OR =1.61, 95% CI 1.05–2.47, *P* = 0.028). In subgroup analyses, individuals were stratified by age, sex, BMI, ACS type, hypertension, and dyslipidemia, showing that there were no significant interactions between the FT3/FT4 ratio and all subgroups for MACCE. In addition, the FT3/FT4 ratio performed better on ROC analyses for cardiac death prediction [area under the curve (AUC), 0.738].

**Conclusion:**

A reduced level of FT3/FT4 ratio was a potential marker of poor prognosis in euthyroid patients with ACS and diabetes after PCI.

## Introduction

Recent research studies show that more than seven million people worldwide are newly diagnosed with acute coronary syndromes (ACS) each year, and about 5% of people with ACS die before they are discharged from the hospital (1, 2). In patients with ACS, the risk of all-cause in-hospital death of patients with diabetes is twice that of patients without diabetes. Moreover, patients with ACS with diabetes have worse long-term prognosis than patients with ACS alone. The risk of major adverse cardiac and cerebrovascular events (MACCE) was 1.5 times that of those without diabetes, and long-term mortality was 50% higher in ACS patients with diabetes than in non-diabetic patients (3, 4). Studies also found that patients with diabetes have a higher ratio of high-risk plaques (or vulnerable plaques) in culprit lesions and non-culprit lesions for ACS than those without diabetes, the same as a worse long-term prognosis (5, 6).

Previous studies have also found that coronary heart disease and diabetes are closely related to thyroid hormone. Hyperthyroidism, hypothyroidism, and subclinical thyroid diseases are related to MACCE of cardiovascular system diseases (7–10). The potential mechanisms may include cellular inflammatory responses, vascular endothelial cell damage, dyslipidemia, atherosclerosis, and cardiac dysfunction (11, 12). Recently, the ratio of free triiodothyronine (FT3) to free thyroxine (FT4), as an indirect index reflecting the conversion of T4 to T3 and the peripheral deiodinase activity (13, 14), can better reflect the real thyroid function status than the serum levels of thyroid function indicators, including thyroid-stimulating hormone (TSH), triiodothyronine (TT3), thyroxine (TT4), FT3, and FT4. Moreover, it has been gradually confirmed that it is associated with the risk of hypertension, cardiovascular disease, pre-diabetes, diabetes, metabolic syndrome, renal insufficiency, and residual cholesterol levels in euthyroid patients (9–12, 15, 16). A lower FT3/FT4 ratio is related to an adverse prognosis in different cohorts with myocardial infarction with nonobstructive coronary arteries (MINOCA) and CAD (17). However, the correlation between the FT3/FT4 ratio and long-term prognosis in euthyroid patients with ACS and diabetes after PCI remains unclear. Therefore, the objective of this prospective study is to assess the potential relationship between the FT3/FT4 ratio and poor prognosis in euthyroid patients with ACS and diabetes after PCI. It is expected to provide some clinical reference value for the prevention, screening, and improvement of the prognosis of the disease.

## Method

### Study population

This prospective cohort study enrolled 1,786 euthyroid patients with ACS and diabetes who successfully underwent PCI, and only the culprit lesion was performed at Beijing Anzhen Hospital from August 2021 to April 2022. Patients who were classified under the following criteria were excluded: (1) infectious disease, (2) blood diseases, (3) malignant neoplasms, (4) liver and kidney diseases, (5) pregnant or lactating women, (6) other drugs that can affect thyroid function, such as amiodarone, (7) patients who had a history of thyroid disease, taking thyroid drugs, or thyroid surgery and who have not received thyroid function tests, and (8) history of hypothalamus or pituitary disease. This prospective cohort study follows the principles of the Declaration of Helsinki. All participants signed a written informed consent. Blood samples were collected in the morning after overnight fasting and tested on the same day using standard laboratory methods in the central laboratory. The thyroid function profile was measured quantitatively by direct chemiluminescence method (ADVIA Centaur, Siemens, USA), including serum FT3, FT4, and TSH, which was performed in the morning after admission. The normal reference intervals of our hospital were as follows: FT3, 3.28 to 6.47 pmol/L; FT4, 7.64 to 16.03 pmol/L; and TSH, 0.49 to 4.91 mIU/L. Euthyroid is defined as circulating TSH level; FT3 and FT4 are within the reference range. The WHO hypertension guidelines define the initial diagnosis of hypertension as systolic blood pressure greater than 140 mmHg or diastolic blood pressure of not less than 90 mmHg (18). Diabetes was defined as fasting blood glucose ≥7.0 mmol/L, 2-h plasma glucose ≥11.1 mmol/L, or having a diabetic history (19). Dyslipidemia was diagnosed by medical history or having low-density lipoprotein (LDL) cholesterol ≥3.4 mmol/L, high-density lipoprotein cholesterol<1.0 mmol/L, or triglyceride ≥1.7 mmol/L (20).

### Study endpoints and follow-up

Clinical follow-up was conducted by a skilled clinician through outpatient or telephone visits. All patients were followed for an average of 16 months. A group of clinical cardiologists made a joint decision on the classification of the endpoint events. The primary endpoint was major adverse cardiac and cerebrovascular events (MACCE), including all-cause death, nonfatal myocardial infarction (MI), revascularization, and nonfatal stroke. The secondary endpoints included cardiac death, hospitalization for heart failure (HF), unstable angina (UA), and each component of MACCE. All deaths are considered cardiac deaths unless a non-cardiac cause is established. Revascularization was defined as unplanned revascularization (PCI or CABG) associated with the target lesion. A stroke is defined as any sudden, focal, or global neurological impairment caused by cerebral ischemia or bleeding that lasts for more than 24 h or results in death (with CT or MRI imaging evidence).

### Statistical analysis

Continuous variables that fit the normal distribution were presented as mean ± standard deviation, and correlations between continuous variables were evaluated using Pearson product difference analysis (Pearson *r*) or Spearman rank correlation analysis (Spearman *r*). Continuous variables that are not normally distributed are described by the median of the quartile interval. Categorical variables were represented by percentiles, and correlations were analyzed using chi-square tests. Kaplan–Meier method was used to construct a survival curve, and log-rank test was used to compare the differences between groups. Univariate and multivariate Cox proportional hazard regression were used to evaluate the relationship between FT3/FT4 ratio levels and prognosis. The screening of confounding factors in the multivariate Cox proportional hazards model was mainly based on statistically significant clinical variables after univariate Cox regression analyses (*P* < 0.05). Then, we also incorporated confounding variables (age, gender, and BMI) that may affect the clinical prognosis of ACS into the model to obtain more accurate HR results as much as possible. Cox model 1 was the unadjusted model; model 2 was adjusted by confounding factors including age and sex; and model 3 was further adjusted for BMI, ACS classification (UA or NSTEM or STEMI), hypertension, and dyslipidemia. The area under the curve (AUC) is defined using receiver operating characteristic curve (ROC) analysis. A two-tailed *P*-value of <0.05 was statistically significant. R version 4.2.3. and SPSS version 25.0 (SPSS, Chicago, IL, USA) were used to perform statistical analysis.

## Results

### Patients’ baseline characteristics

**Figure 1:**
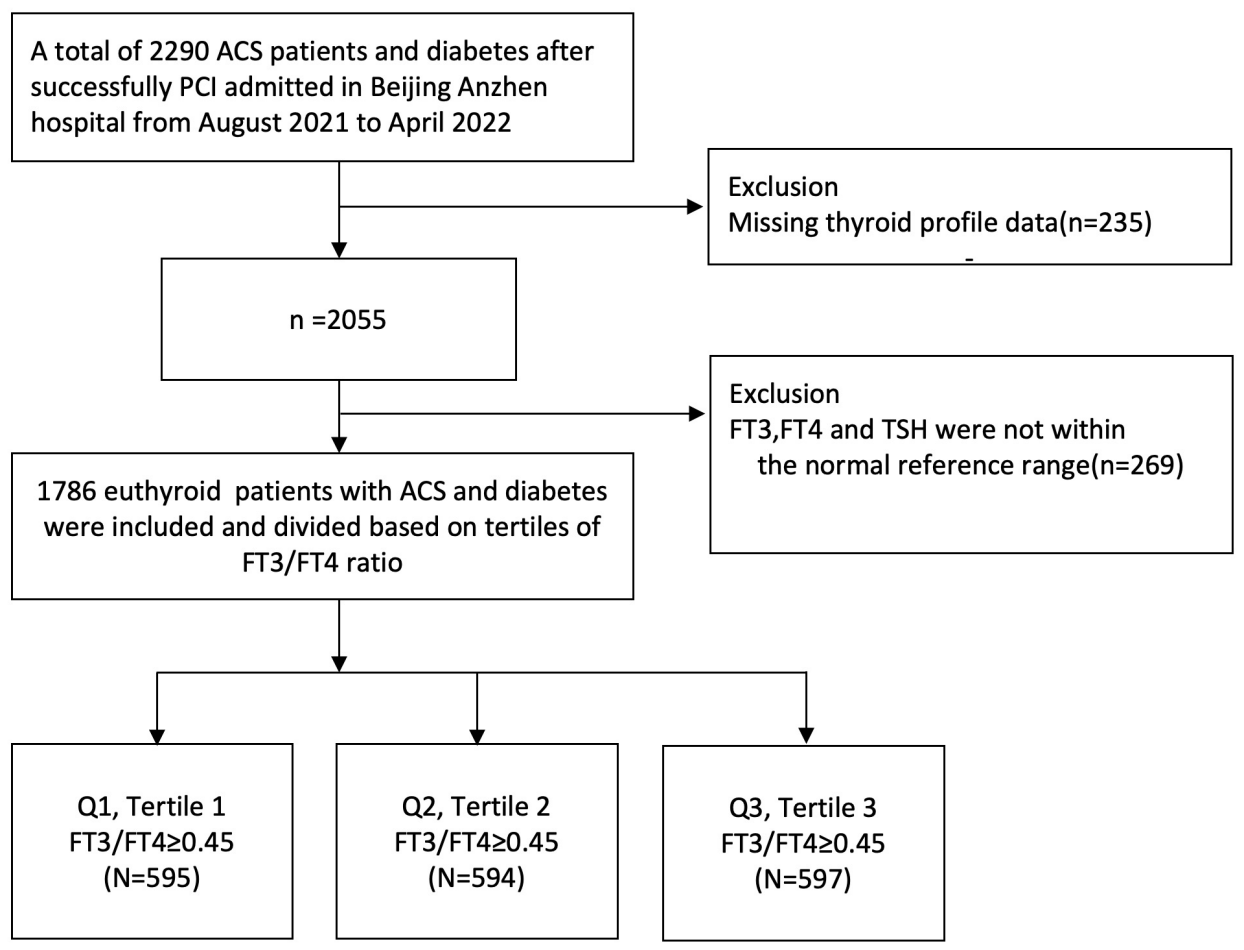
Study flowchart.

The patients were divided into three groups according to the decrease in FT3/FT4 ratio (tertile 1: FT3/FT4 ≥0.45, *n* = 595; tertile 2: 0.39 ≤ FT3/FT4 <0.45; *n* = 594; tertile 3: FT3/FT4 <0.39; *n* = 597) (**Figure 1**). In **Table 1**, the proportions of patients who were older, had hypertension, and were female are significantly higher in the lowest FT3/FT4 tertile, as well as the level of BMI and HbA1c (all *p* < 0.05). The three groups did not exhibit any statistically significant differences in terms of MI, PCI, CABG, LDL-C, LVEF, creatinine levels, and drug utilization (all *p* > 0.05). We also collected and analyzed the PCI data of patients in the three groups and found that there was no statistical difference in terms of multi-vessel lesions, lesion types, number of stents, and so on (**Supplementary Table S1**).

**Table 1:** Baseline characteristics of the study population grouped by FT3/FT4 ratio.

	Q1 (*n* = 595) (fT3/fT4 ≥ 0.45)	Q2 (*n* = 594) (0.39 ≤ fT3/fT4 < 0.45)	Q3 (*n* = 597) (fT3/fT4 < 0.39)	*P*
Age, year	58.15 ± 9.27	60.37 ± 9.40	63.35 ± 9.69	<0.001
Male	468(78.7)	433 (72.9)	386 (64.7)	<0.001
Hypertension	418 (70.3)	416 (70.0)	461 (77.2)	0.007
Diabetes	595 (100)	594 (100)	597 (100)	1.0
Hyperlipidemia	517 (86.9)	512 (86.2)	481 (80.6)	0.004
Current smoking	167 (28.1)	194 (32.7)	152 (25.5)	0.508
Prior-PCI	229 (38.5)	208 (35.0)	196 (32.8)	0.120
Prior-MI	103 (17.3)	90 (15.2)	95 (15.9)	0.590
Prior-CABG	16 (2.7)	17 (2.9)	16 (2.7)	0.977
Prior stroke	41 (6.9)	49 (8.2)	65 (10.9)	0.045
PAD	23 (3.9)	16 (2.7)	23 (3.9)	0.448
LVEF, %	61.09 ± 7.41	61.33 ± 6.99	59.68 ± 9.23	0.322
BMI, kg/m^2^	26.70 ± 3.32	26.06 ± 3.39	25.78 ± 2.94	<0.001
CR, mmol/L	81.43 ± 40.04	82.38 ± 22.60	85.36 ± 65.89	0.124
eGFR, ml/min/1.73 m^2^	86.02 ± 17.37	88.45 ± 17.77	82.24 ± 20.61	0.430
GLU, mmol/L	8.31 ± 3.13	8.29 ± 3.57	8.69 ± 9.43	0.380
HbA1C, %	7.38 ± 1.24	7.35 ± 1.22	7.39 ± 1.21	0.029
TG, mmol/L	1.94 ± 1.18	1.86 ± 1.78	1.68 ± 1.11	0.578
TC, mmol/L	4.12 ± 1.10	4.02 ± 1.06	3.95 ± 1.03	0.695
HDL, mmol/L	1.00 ± .022	1.01 ± 0.25	1.04 ± 0.26	0.002
LDLC, mmol/L	2.22 ± 0.86	2.16 ± 0.83	2.08 ± 0.81	0.925
Hs-CRP	2.65 ± 5.62	2.46 ± 4.48	3.78 ± 9.41	0.312
TT4, nmol/L	111.79 ± 20.57	117.37 ± 21.34	123.66 ± 21.30	0.178
TT3, nmol/L	1.51 ± 0.29	1.42 ± 0.27	1.48 ± 5.33	0.702
TSH, mIU/L	2.01 ± 1.00	1.91 ± 0.93	1.83 ± 0.95	0.558
FT3, pmol/L	4.98 ± 0.51	4.69 ± 0.45	4.33 ± 0.48	0.002
FT4, pmol/L	9.99 ± 1.12	11.26 ± 1.08	12.58 ± 1.44	0.414
ACS				0.006
UAP	534 (89.7)	521 (87.7)	495 (82.9)	
NSTEMI	44 (7.4)	45 (7.6)	64 (10.7)	
STEMI	17 (2.9)	28 (4.7)	38 (6.4)	
SGLT2 inhibitor	283 (47.6)	305 (51.3)	293 (49.1)	0.303
Canagliflozin	24 (4.0)	23 (3.9)	25 (4.2)	0.652
Empagliflozin	24 (4.0)	21 (3.5)	21 (3.5)	0.504
Dapagliflozin	235 (39.5)	261 (43.9)	247 (41.4)	0.332
Metformin	290 (48.7)	314 (52.9)	302 (50.6)	0.224
Alpha glucosidase inhibitor	180 (30.3)	192 (32.2)	202 (33.8)	0.172
Sulfonylurea	78 (13.1)	80 (13.5)	93 (15.6)	0.843
No-sulfonylurea	12 (2.0)	12 (2.0)	14 (2.3)	0.238
Thiazolidinedione	7 (1.2)	10 (1.7)	8 (1.3)	0.434
DDP4 inhibitor	53 (8.9)	69 (11.6)	59 (9.9)	0.570
GLP1 receptor agonist	22 (3.7)	16 (2.7)	11 (1.8)	0.291
Insulin	165 (27.7)	154 (25.9)	189 (31.7)	0.189
Aspirin	584 (98.2)	584 (98.3)	585 (98.0)	0.517
Clopidogrel	366 (61.5)	374 (63.0)	392 (65.7)	0.355
Ticagrelor	229 (38.5)	220 (37.0)	205 (34.3)	0.355
Statin	593 (99.7)	593 (99.8)	594 (99.5)	1.0
ACEI/ARB	222 (37.3)	247 (41.6)	266 (44.6)	0.715
β-receptor blockers	392 (65.9)	402 (67.7)	397 (66.5)	0.801
CCB	216 (36.3)	230 (38.7)	221 (37.0)	0.024

Values are presented as mean standard deviation or number (%). eGFR was calculated according to the CKD-EPI formula.

PCI, percutaneous coronary intervention; MI, myocardial infarction; CABG, coronary artery bypass grafting; HF, heart failure; CKD, chronic kidney dysfunction; PAD, peripheral artery disease; LVEF, left ventricular ejection fraction; BMI, body mass index; CR, creatinine; eGFR, estimated glomerular filtration rate; GLU, glucose; TG, total cholesterol; TC, total triglycerides; LDLC, low-density lipoprotein cholesterol; FT3, free triiodothyronine; FT4, free thyroxine; TSH, thyroid-stimulating hormone; T3, total triiodothyronine; T4, total thyroxine; ACS, acute coronary syndrome; UAP, unstable angina pectoris; STEMI, ST-elevation myocardial infarction; NSTEMI, non-elevation myocardial infarction; ACEI, angiotensin-converting enzyme inhibitors; DDP4, dipeptidyl peptidase 4; ARB, angiotensin II receptor blockers; CCB, calcium channel blocker; GLP-1, glucagon-like peptide-1.

### Relationship between the FT3/FT4 index and clinical endpoint events

**Figure 2:**
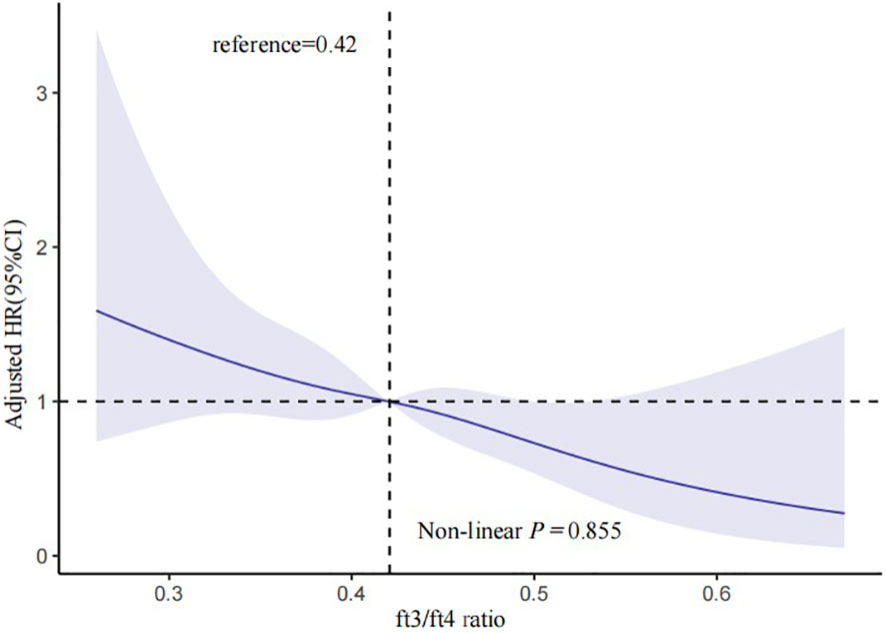
Multivariable-adjusted HR for MACCE based on restricted cubic splines for the FT3/FT4 ratio. The blue lines represent references for HR, and the blue areas represent 95% CI. HR was adjusted for age, sex, BMI, ACS classification, hypertension, and dyslipidemia in the multivariate model. MACCE, major adverse cardiac and cerebrovascular events; HR, hazard ratio; CI, confidence interval; BMI, body mass index; ACS, acute coronary syndrome.

**Figure 3:**
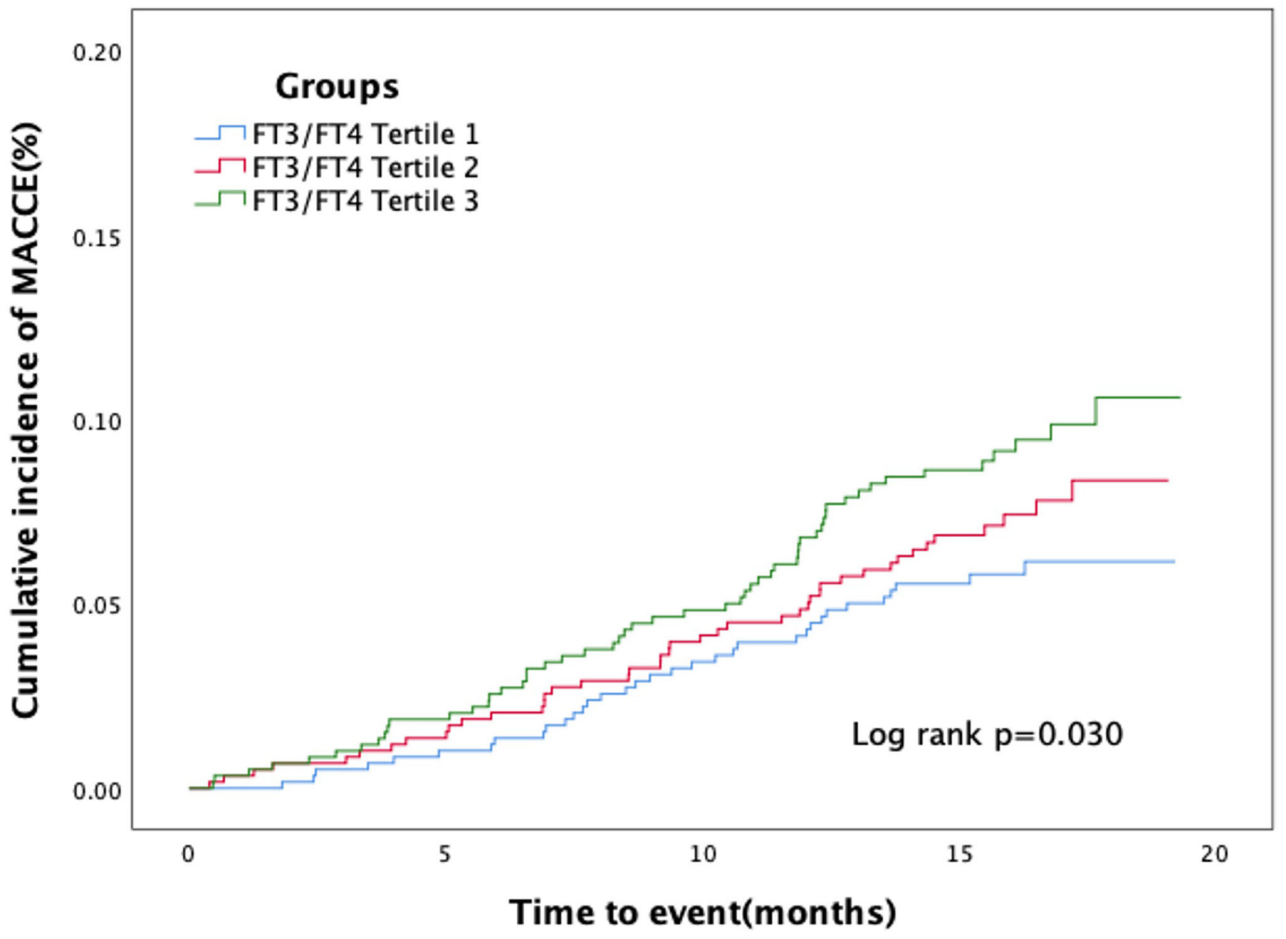
Kaplan–Meier curves for major adverse cardiac and cerebrovascular events according to tertiles of FT3/FT4 ratio.

**Figure 4:**
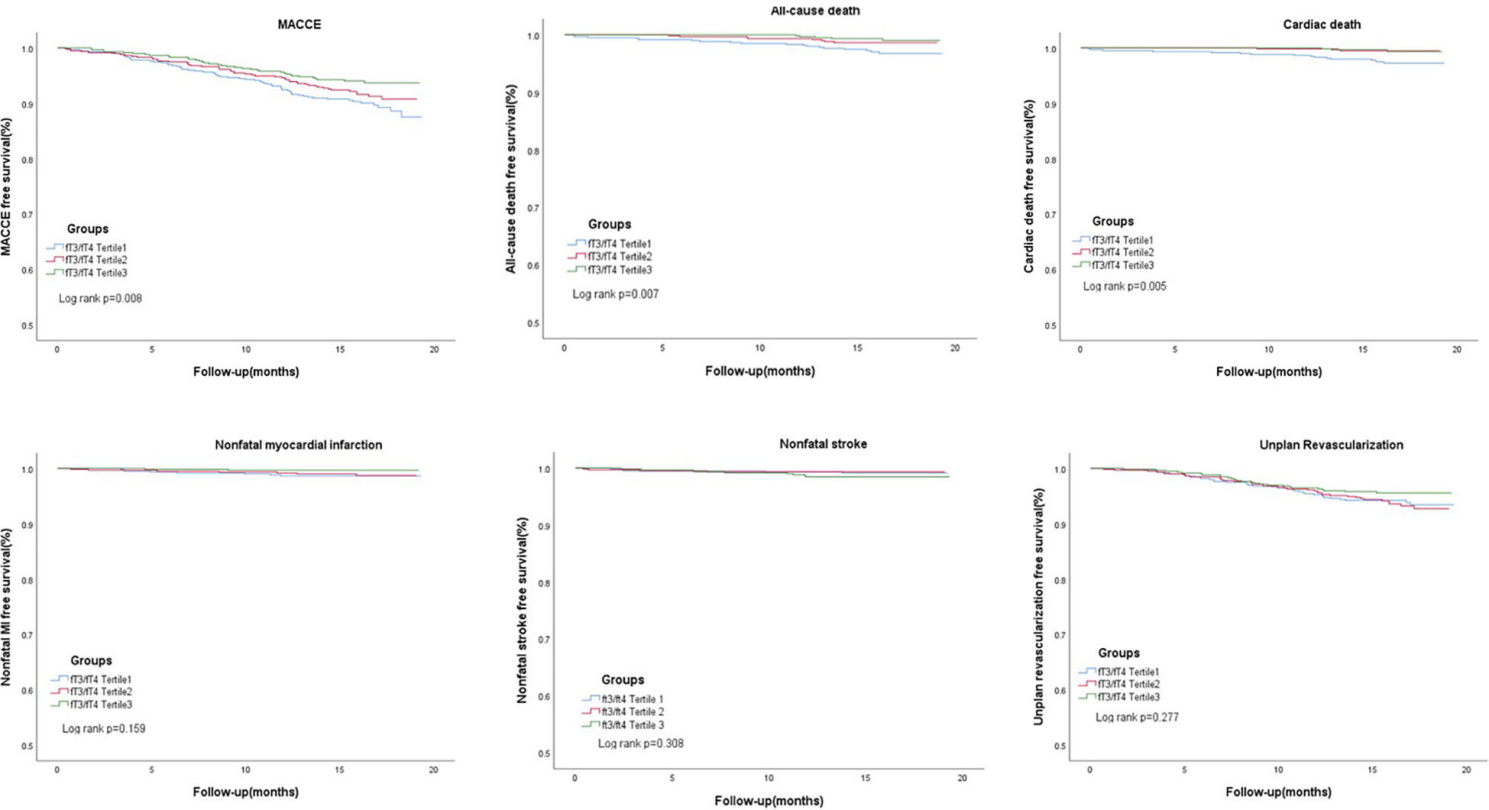
Cumulative survival curves for the primary and secondary endpoints in the three groups.

In **Table 2**, 148 euthyroid patients with ACS developed MACCE (31 died, 17 had nonfatal MI, 18 had a nonfatal stroke, and 100 suffered from revascularization) during the median follow-up time of 16 months; meanwhile, 125 patients were hospitalized for unstable angina, and 20 patients were hospitalized for heart failure. The group with a lower FT3/FT4 ratio had a significantly higher incidence of MACCE, and it increased with decreasing FT3/FT4 tertiles (6.1%, 8.4%, and 10.4%; *p* = 0.025). Patients with lower levels of FT3/FT4 ratio were associated with a significantly higher incidence of long-term MACCE, cardiac death, all-cause death, and hospitalization for HF (all *P* < 0.05), while the rate of nonfatal MI, nonfatal stroke, revascularization, and hospitalization for UA was not statistically different between the three groups (**Table 2**). **Figure 2** presents the results of the restricted cubic splines and shows a dose–response relationship between the FT3/FT4 ratio and risk of MACCE (non-linear *P* = 0.855). In addition, the Kaplan–Meier curve shows that the cumulative incidence of MACCE was significantly higher in patients with a lower FT3/FT4 ratio tertile and increased incrementally across tertiles of the FT3/FT4 ratio (log-rank *p* = 0.030) (**Figure 3**). The cumulative survival curves of the primary and secondary endpoints for the overall population also showed similar results (log-rank *P* < 0.05 for MACCE, cardiac death, and all-cause death) (**Figure 4**).

**Table 2:** Clinical outcomes in ACS patients based on tertiles of FT3/FT4 ratio.

Events	Q1 (fT3/fT4 ≥ 0.45) (*n* = 595)	Q2 (0.39 ≤ fT3/fT4 < 0.45) (*n* = 594)	Q3 (fT3/fT4 < 0.39) (*n* = 597)	*P*
MACCE	36 (6.1%)	50 (8.4%)	62 (10.4%)	0.025
All-cause death	5 (0.8%)	8 (1.3%)	18 (3.0%)	0.011
Cardiac death	3 (0.5%)	3 (0.5%)	15 (2.5%)	0.001
Nonfatal MI	2 (0.3%)	7 (1.2%)	8 (1.3%)	0.160
Nonfatal stroke	5 (0.8%)	4 (0.7%)	9 (1.5%)	0.312
Revascularization	26 (4.4%)	38 (6.4%)	36 (6.0%)	0.269
Hospitalization for HF	5 (0.8%)	2 (0.3%)	13 (2.2%)	0.008
Hospitalization for UA	42 (7.1%)	45 (7.6%)	38 (6.4%)	0.713

**Figure 5:**
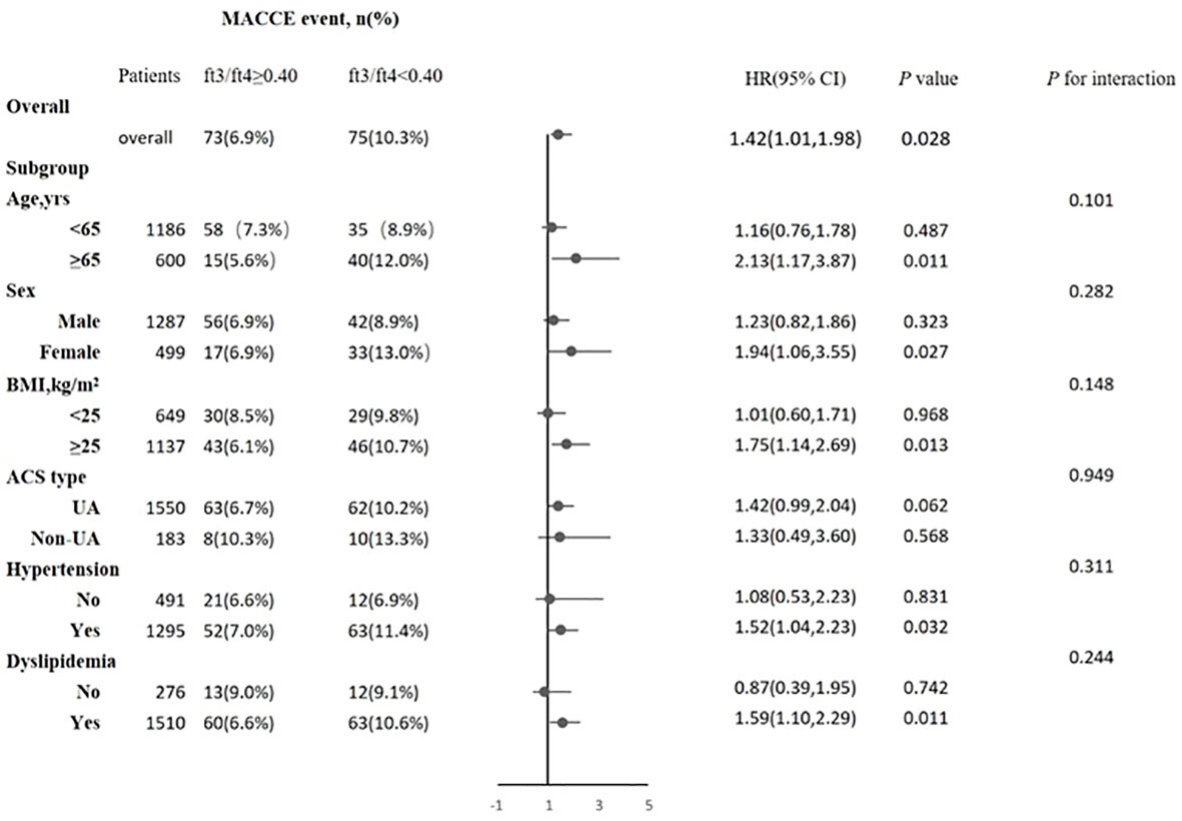
Association between FT3/FT4 ratio and the risk of major adverse cardiac and cerebrovascular events (MACCE) in overall and subgroups. Subgroup analysis showing the incidence and risk of MACCE in patients with FT3/FT4 ratio above and below the cut-off value of 0.40, which was identified with maximum Youden index in all ACS patients for MACCE prediction. The hazard ratio (HR) was calculated by multivariate Cox regression analysis. The vertical dotted line indicated the HR value of 1. BMI, body mass index; UA, unstable angina, CI, confidence interval.

Cox regression analyses were used to estimate the association between the FT3/FT4 ratio and outcomes. The screening of confounding factors in the multivariate Cox proportional hazards model was mainly based on statistically significant clinical variables after univariate Cox regression analyses, and the results showed four statistically significant variables [BMI, ACS classification (UA or NSTEM or STEMI), hypertension, and dyslipidemia]. Then, we also incorporated confounding variables (age and gender) that may affect the clinical prognosis of ACS into the model to obtain more accurate HR results as much as possible. In our study, renal failure, anemia, LVEF, and so on were not statistically significant in the univariate Cox regression. Multivariate Cox proportional hazard regression analysis showed that the FT3/FT4 ratio remained significant after adjusting for confounding factors, regardless of whether they were categorical or continuous variables. Compared with patients in the highest FT3/FT4 tertile, the increased risk of MACCE after adjustment for age and sex was 1.40 (95% CI: 0.90–2.12) in the middle (Q2) and 1.71 (95% CI: 1.10–2.65) in the lowest tertile (Q3), and after multivariate adjustment it was 1.38 (95% CI: 0.90–2.12) in the middle (T2) and 1.61 (95% CI: 1.05–2.47) in the lowest tertile (Q3), respectively (**Table 3**). At ROC analysis, the cutoff of FT3/FT4 that maximized the sensitivity and specificity for MACCE prediction in all patients was identified as 0.40. In total, 1,059 patients (59.3%) had a ratio above the cutoff value. The incidence of MACCE was 6.9% and 10.3% (*p* < 0.001) in patients with FT3/FT4 above and below the cutoff, respectively (HR 1.42, 95% CI: 1.02–1.99, *p* = 0.03) (**Figure 5**).

**Table 3:** Risk of primary and secondary outcomes according to FT3/FT4 ratio in the three Models.

	Model 1		Model 2		Model 3	
Outcomes	HR (95% CI)	*P*-value	HR (95% CI)	*P*-value	HR (95% CI)	*P*-value
FT3/FT4 ratio as a categorical variable						
MACCE						
Q1	Reference	–	Reference	–	Reference	–
Q2	1.43 (0.92, 2.23)	0.117	1.40 (0.90, 2.19)	0.141	1.378 (0.90, 2.12)	0.146
Q3	1.80 (1.17, 2.76)	**0.007**	1.71 (1.10, 2.65)	**0.017**	1.61 (1.05, 2.47)	**0.028**
*P* for trend	0.171		0.260		0.338	
All-cause death						
Q1	Reference	–	Reference	–	Reference	–
Q2	1.61 (0.52, 4.96)	0.405	1.42 (0.46, 4.42)	0.541	1.40 (0.46, 4.32)	0.554
Q3	3.67 (1.35, 9.95)	**0.011**	2.66 (0.95, 7.44)	0.063	2.44 (0.87, 6.83)	0.091
*P* for trend	0.023		0.112		0.146	
Cardiac death						
Q1	Reference	–	Reference	–	Reference	–
Q2	1.00 (0.20, 4.98)	0.998	0.89 (0.18, 4.47)	0.889	0.89 (0.18, 4.43)	0.885
Q3	5.09 (1.47, 17.67)	**0.010**	3.75 (1.04, 13.51)	0.044	3.44 (0.95, 12.49)	0.060
*P* for trend	0.002		0.012		0.016	
Nonfatal MI						
Q1	Reference	–	Reference	–	Reference	–
Q2	3.54 (0.73, 17.09)	0.116	3.41 (0.70, 16.60)	0.129	3.35 (0.69, 16.22)	0.133
Q3	4.03 (0.85, 19.04)	0.079	3.80 (0.78, 18.43)	0.098	3.38 (0.69, 16.57)	0.134
*P* for trend	0.668		0.747		0.011	
Nonfatal stroke						
Q1	Reference	–	Reference	–	Reference	–
Q2	0.80 (0.21, 3.00)	0.740	0.77 (0.20, 2.89)	0.696	0.79 (0.21, 2.98)	0.731
Q3	1.806 (0.602, 5.421)	0.292	1.69 (0.55, 5.26)	0.362	1.80 (0.58, 5.56)	0.310
*P* for trend	0.145		0.169		0.154	
Unplan revascularization						
Q1	Reference	–	Reference	–	Reference	–
Q2	1.50 (0.90, 2.50)	0.124	1.52 (0.91, 2.55)	0.112	1.50 (0.91, 2.48)	0.112
Q3	1.40 (0.84, 2.36)	0.199	1.44 (0.85, 2.46)	0.177	1.42 (0.84, 2.39)	0.187
*P* for trend	0.895		0.906		0.847	
FT3/FT4 ratio as a continuous variable						
MACCE	0.03 (0.00–0.34)	**0.004**	0.04 (0.00–0.49)	**0.011**	0.05 (0.01–0.57)	**0.016**
All–cause death	0.00 (0.00–0.03)	**0.001**	0.00 (0.00–0.36)	**0.021**	0.00 (0.00–0.50)	**0.028**
Cardiac death	0.00 (0.00–0.00)	**0.000**	0.00 (0.00–0.03)	**0.003**	0.00 (0.00–0.04)	**0.004**
Nonfatal MI	0.00 (0.00–1.23)	0.057	0.00 (0.00–1.86)	0.072	0.00 (0.00–5.70)	0.134
Nonfatal stroke	0.01 (0.00–8.67)	0.180	0.01 (0.00–13.37)	0.216	0.01 (0.00–11.08)	0.192
Unplan revascularization	0.17 (0.01–2.74)	0.211	0.15 (0.01–2.65)	0.196	0.15 (0.01–2.75)	0.203

The patients were divided into three groups according to the decrease of FT3/FT4 ratio (Q1, tertile 1: FT3/FT4 ≥ 0.45, *n* = 595; Q2, tertile 2:0.39 ≤ FT3/FT4 < 0.45; *n* = 594; Q3, tertile 3: FT3/FT4 < 0.39; 2.28, *n* = 597). Model 1 was the unadjusted model. Model 2 included sex and age. Model 3 included age, sex, BMI, ACS classification (UA or NSTEM or STEMI), hypertension, and dyslipidemia in the multivariate Cox analysis.

Bold represents P value <0.05.

### Subgroup analysis

The association between the FT3/FT4 ratio and MACCE was examined in the subgroup analysis according to age (>65 or ≤65 years), sex (male or female), BMI (≥25.0 or <25.0 kg/m^2^), ACS type (UA or non-UA), hypertension (yes or no), and hyperlipidemia (yes or no). There was no statistically significant interaction between age, sex, BMI, ACS type, hypertension, hyperlipidemia, and FT3/FT4 ratio (all *P*-values for interaction ≥0.05). A lower FT3/FT4 (<0.40) remained a risk factor in subsets of patients stratified by age, sex, BMI, ACS type, hypertension, and hyperlipidemia (all *p* < 0.05) (**Figure 5**), suggesting that the prognostic effect of FT3/FT4 ratio was not affected by clinically relevant demographic or traditional risk factors.

### Predictive value of the FT3/FT4 ratio for MACCE

**Figure 6:**
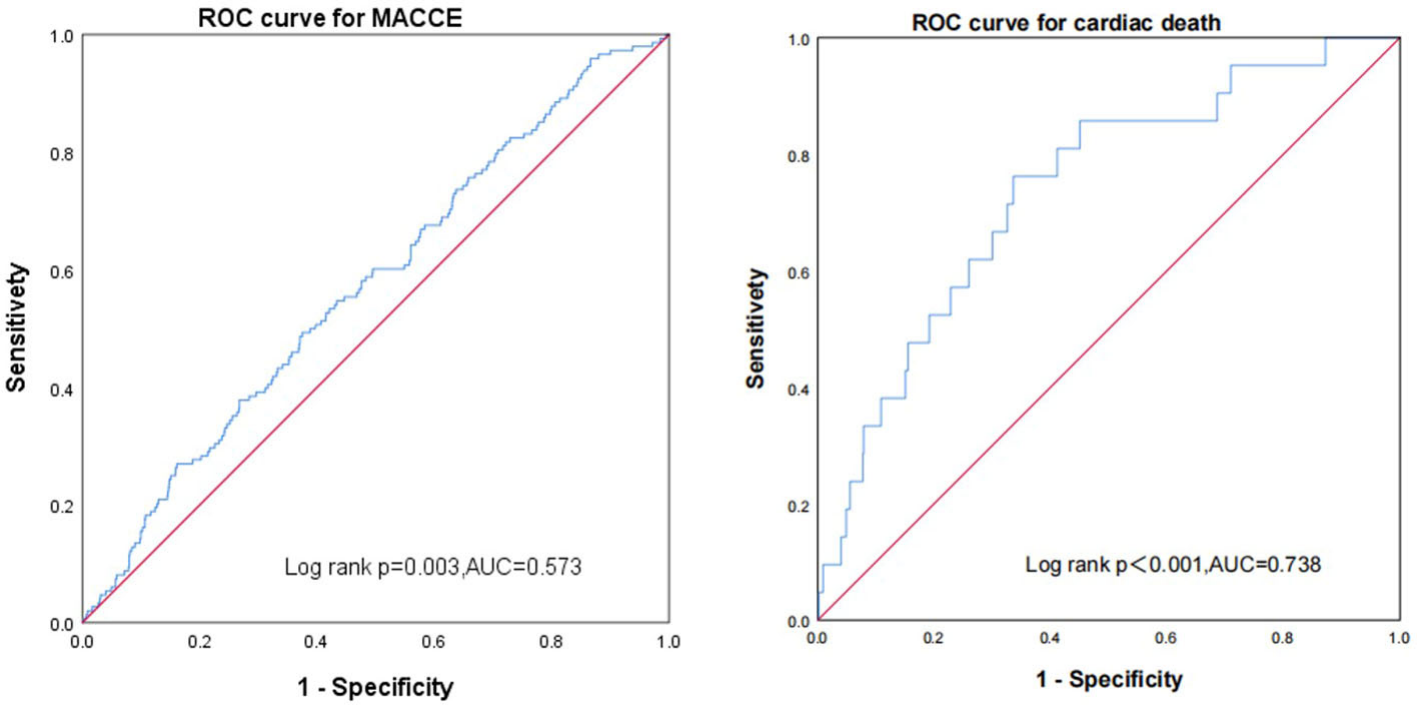
Receiver operating characteristic curves showing the predictive value of the FT3/FT4 ratio using Cox regression in major adverse cardiac and cerebrovascular events and cardiac death.

Receiver operating characteristic curves show the predictive value of the FT3/FT4 ratio (AUC 0.573, 95% CI: 0.526–0.620, *p* < 0.003). The FT3/FT4 ratio showed a better value of cardiac death than MACCE in euthyroid patients with ACS combined with diabetes after PCI (AUC: 0.738, 95% CI: 0.635–0.842, *p* < 0.001) (**Figure 6**).

## Discussion

This study is the first prospective cohort study that examined the association between FT3/FT4 ratio and 16-month prognosis in euthyroid patients with ACS and diabetes successfully undergoing PCI. It demonstrated that (1) a decreased peripheral sensitivity to the thyroid hormone index of FT3/FT4 ratio was independently associated with an increased risk of 16-month MACCE and (2) the FT3/FT4 ratio might be a valuable prognostic marker for the identification and management of high-risk individuals in this specific patient population.

The prevalence rate of ACS and diabetes was as high as 24%–30%, and the risk of in-hospital and long-term major adverse cardiovascular events and complications was significantly higher than that of patients without diabetes (5, 6). Some previous retrospective studies with a small sample have found that the proportion of ACS patients with thyroid dysfunction is about 20%–30% (7, 21). As is known to all, the rates of heart failure, nonfatal reinfarction, unplanned repeat revascularization, and all-cause or cardiac mortality are high in ACS patients with thyroid dysfunction, including hyperthyroidism, hypothyroidism, and even subclinical thyroid disease (7, 8, 22). However, in euthyroid patients with normal serum thyroid function indexes, it is very critical to evaluate real thyroid function, identify thyroid dysfunction, and explore its relationship with the prognosis of ACS with diabetes (23).

Previous studies have found that a single measure of thyroid function does not adequately explain the relationship between the thyroid system and cardiovascular disease risk and show inconsistent results (22, 24, 25). In recent studies, indices of thyroid hormone sensitivity have been further explored in association with the risks of hypertension, cardiovascular disease, pre-diabetes, diabetes, metabolic syndrome, frailty, renal insufficiency, and residual cholesterol level in euthyroid patients (9–12, 15, 16, 26). Moreover, a higher FT3/FT4 value is correlated with a greater sensitivity of the peripheral tissue to thyroid hormone (11, 27). Brozaitiene et al. demonstrated that the FT3/FT4 ratio could serve as a valuable predictor of long-term outcomes of CAD patients undergoing PCI. However, this study did not measure the FT3 levels and other thyroid hormones during the acute stage of coronary syndromes (22). Yu et al. showed that the FT3/FT4 ratio was an independent predictor of 12-month all-cause mortality in euthyroid patients with AMI undergoing PCI, and its prognostic performance was comparable to that of the GRACE score. Nevertheless, some patients’ thyroid function was tested following exposure to iodinated contrast media due to emergency PCI, which may affect the resulting authenticity (28). Gao et al. first found that a decreased FT3/FT4 ratio was an independent predictor of poor outcomes in euthyroid patients with MINOCA (17). However, the correlation between sensitivity to thyroid hormone and long-term prognosis in euthyroid patients with ACS and diabetes after PCI remains unclear.

The present study, for the first time, found that the FT3/FT4 ratio was associated with 16-month MACCE [MACCE for the lower FT3/FT4 ratio group (FT3/FT4 <0.40) vs. the higher FT3/FT4 ratio group (FT3/FT4 ≥0.40): 10.3% vs. 6.9%, log-rank test: *p* = 0.028] in euthyroid patients with ACS complicated with diabetes undergoing PCI. Multivariate Cox proportional hazard regression analysis showed that the FT3/FT4 ratio remained significant after adjusting for confounding factors, regardless of whether they were categorical or continuous variables. The results of the subgroup analysis demonstrated that the prognostic impact of the FT3/FT4 ratio remained consistent across clinically relevant demographic and traditional risk factors. In summary, the FT3/FT4 ratio serves as a valuable clinical indicator for predicting long-term adverse outcomes in euthyroid patients with ACS and diabetes undergoing PCI, and it aids in effective risk stratification for these individuals.

Most studies on the mechanism of thyroid dysfunction leading to a poor prognosis of cardiovascular diseases focus on dyslipidemia caused by hypothyroidism (29). Other studies have found that dysfunction of endothelial cells, macrophages, and vascular smooth muscle cells (VSMCs) is associated with hypothyroidism (30, 31). However, whether and how hypothyroidism affects inflammation and the immune system in the context of atherosclerosis has not been fully elucidated. The decrease in the FT3/FT4 ratio reflects the abnormal conversion of T4 to T3 during acute or chronic myocardial injury (26, 32). Previous studies have found that low T3 syndrome may represent a hormone homeostasis escape response, which means that it is a beneficial and physiological adaptation mechanism by reducing myocardial metabolic demand and preventing arrhythmia in the early stress stage of acute ischemic events (33, 34). However, recent studies found that a lower FT3/FT4 ratio has worse effects on the cardiovascular system by reducing cardiac contractility and increasing vascular resistance, and a persisting downregulated thyroid system after ACS might become maladaptive because of the loss of the positive effects of T3 on the cardiovascular system (35). However, despite this relationship, there are further experimental and randomized controlled investigations to confirm the value and underlying biological mechanisms between the FT3/FT4 ratio and poor prognosis in this population.

On the other hand, there are several possible mechanisms involved in thyroid hormones affecting glucose metabolism as follows: (a) reduced half-life of insulin caused by an increasing rate of degradation and enhancing the release of biologically inactive insulin precursors in hyperthyroidism (36, 37), (b) increased intestinal glucose absorption mediated by excess thyroid hormones (38, 39), and (c) thyroid hormones raise GLUT-2 (the glucose transporter in the liver), leading to increased glucose output and abnormal glucose metabolism (40). Although the relationship between FT3/FT4 ratio and diabetes has been demonstrated, the specific regulatory mechanisms remain unknown (16).

This study has several limitations. Firstly, this study was a single-center observational study with potential confounding factors and selection bias. Secondly, as recommended by the guidelines, thyroid function tests are not repeated within 2–12 weeks to rule out transient forms of thyroid dysfunction (41). Thirdly, thyroid function was not further assessed during follow-up to identify new thyroid diseases and explore the relationship between FT3/FT4 ratio variation rate and outcome. Finally, there were relatively few AMI patients in ACS patients undergoing PCI in this study.

## Conclusions

The FT3/FT4 ratio demonstrated an independent association with MACCE in euthyroid patients with ACS and diabetes undergoing PCI. Therefore, it is recommended to monitor the FT3/FT4 ratio while intensifying the management of traditional cardiovascular risk factors.

## Data availability statement

The raw data supporting the conclusions of this article will be made available by the authors, without undue reservation.

## Ethics statement

The studies involving humans were approved by Ethics Committee of Beijing Anzhen Hospital and obeyed the principles of the Declaration of Helsinki. The studies were conducted in accordance with the local legislation and institutional requirements. Written informed consent for participation was not required from the participants or the participants’ legal guardians/next of kin in accordance with the national legislation and institutional requirements.

## Author contributions

SW: Writing – original draft, Conceptualization, Data curation, Formal analysis, Investigation, Methodology, Project administration. YW: Formal analysis, Resources, Writing – original draft. SS: Data curation, Investigation, Writing – original draft. FL: Software, Writing – original draft. WZ: Data curation, Investigation, Writing – original draft. XL: Data curation, Formal analysis, Writing – original draft. MY: Data curation, Methodology, Writing – original draft. YN: Data curation, Investigation, Writing – original draft. XW: Funding acquisition, Supervision, Visualization, Writing – review & editing.

## Supplementary material

Supplementary Table 1 Baseline percutaneous coronary intervention characteristics of the study population grouped by FT3/FT4 ratio.

## References

[1] Reed GW Rossi JE Cannon CP. Acute myocardial infarction. Lancet. (2017) 389:197–210. doi: 10.1016/S0140-6736(16)30677-8 27502078

[2] Virani SS Alonso A Benjamin EJ Bittencourt MS Callaway CW Carson AP. Heart disease and stroke statistics-2020 update: A report from the American heart association. Circulation. (2020) 141:e139–596. doi: 10.1161/CIR0000000000000757 31992061

[3] Zhou M Liu J Hao Y Liu J Huo Y Smit SC Jr. Prevalence and in-hospital outcomes of diabetes among patients with acute coronary syndrome in China: findings from the Improving Care for Cardiovascular Disease in China-Acute Coronary Syndrome Project. Cardiovasc Diabetol. (2018) 17:147. doi: 10.1186/s12933-018-0793-x 30482187 PMC6258152

[4] Gholap NN Achana FA Davies MJ Ray KK Gray L Khunti K. Long-term mortality after acute myocardial infarction among individuals with and without diabetes: A systematic review and meta-analysis of studies in the post-reperfusion era. Diabetes Obes Metab. (2017) 19:364–74. doi: 10.1111/dom.12827 27862801

[5] Steg PG Goldberg RJ Gore JM Fox KAA Eagle KA Flather MD. Baseline characteristics, management practices, and in-hospital outcomes of patients hospitalized with acute coronary syndromes in the Global Registry of Acute Coronary Events (GRACE). Am J Cardiol. (2002) 90:358–63. doi: 10.1016/S0002-9149(02)02489-X 12161222

[6] De Luca G Verdoia M Savonitto S Piatti L Grosseto D Morici N. Impact of diabetes on clinical outcome among elderly patients with acute coronary syndrome treated with percutaneous coronary intervention: insights from the ELDERLY ACS 2 trial. J Cardiovasc Med (Hagerstown). (2020) 21:453–9. doi: 10.2459/JCM.0000000000000978 32355067

[7] Arambam P Kaul U Ranjan P Janardhanan R. Prognostic implications of thyroid hormone alterations in acute coronary syndrome-A systematic review. Indian Heart J. (2021) 73:143–8. doi: 10.1016/j.ihj.2020.11.147 PMC8065368 33865509

[8] Chang X Zhang S Zhang M Wang H Fan C Gu Y. Free triiodothyronine and global registry of acute coronary events risk score on predicting long-term major adverse cardiac events in STEMI patients undergoing primary PCI. Lipids Health Dis. (2018) 17:234. doi: 10.1186/s12944-018-0881-7 30309366 PMC6182867

[9] Sun H Zhu W Liu J An Y Wang Y Wang G. Reduced sensitivity to thyroid hormones is associated with high remnant cholesterol levels in chinese euthyroid adults. J Clin Endocrinol Metab. (2022) 108:166–74. doi: 10.1210/clinem/dgac523 36071542

[10] Mehran L Delbari N Amouzegar A Hasheminia M Tohidi M Azizi F. Reduced sensitivity to thyroid hormone is associated with diabetes and hypertension. J Clin Endocrinol Metab. (2022) 107:167–76. doi: 10.1210/clinem/dgab646 34480566

[11] Laclaustra M Moreno-Franco B Mateo-Gallego JML-BR Guallar-Castillon JACP. Impaired sensitivity to thyroid hormones is associated with diabetes and metabolic syndrome. Diabetes Care. (2019) 42:303–10. doi: 10.2337/dc18-1410 30552134

[12] Yang S Lai S Wang Z Liu A Wang W Guan H. Thyroid Feedback Quantile-based Index correlates strongly to renal function in euthyroid individuals. Ann Med. (2021) 53:1945–55. doi: 10.1080/07853890.2021.1993324 PMC8567884 34726096

[13] Maia AL Goemann IM Souza EL Simone M Wajner M. Deiodinases: the balance of thyroid hormone: type 1 iodothyronine deiodinase in human physiology and disease. J Endocrinol. (2011) 209:283–97. doi: 10.1530/JOE-10-0481 21415143

[14] Bassols J Prats-Puig A Soriano-Rodríguez P García-González MM Reid J Martínez-Pascual M. Lower free thyroxin associates with a less favorable metabolic phenotype in healthy pregnant women. J Clin Endocrinol Metab. (2011) 96:3717–23. doi: 10.1210/jc.2011-1784 21917863

[15] Sun Y Teng D Zhao L Shi X Li Y Shan Z. Impaired sensitivity to thyroid hormones is associated with hyperuricemia, obesity, and cardiovascular disease risk in subjects with subclinical hypothyroidism. Thyroid. (2022) 32:376–84. doi: 10.1089/thy.2021.0500 35078326

[16] Liu B Wang Z Fu J Guan H Lyu Z Wang W. Sensitivity to thyroid hormones and risk of prediabetes: A cross-sectional study. Front Endocrinol (Lausanne). (2021) 12:657114. doi: 10.3389/fendo.2021.657114 34017311 PMC8129566

[17] Gao S Ma W Huang S Lin X Yu M. Predictive value of free triiodothyronine to free thyroxine ratio in euthyroid patients with myocardial infarction with nonobstructive coronary arteries. Front Endocrinol (Lausanne). (2021) 12:708216. doi: 10.3389/fendo.2021.708216 34394005 PMC8356082

[18] Al-Makki A DiPette D Whelton PK Murad MH Mustafa RA Acharya S. Hypertension pharmacological treatment in adults: A world health organization guideline executive summary. Hypertension. (2022) 79:293–301. doi: 10.1161/HYPERTENSIONAHA.121.18192 34775787 PMC8654104

[19] American Diabetes Association. 2. Classification and diagnosis of diabetes: Standards of medical care in diabetes-2018. Diabetes Care. (2018) 41:S13–27. doi: 10.2337/dc18-S002 29222373

[20] Rabar S Harker M N Wierzbicki AS Guideline Development Group. Lipid modification and cardiovascular risk assessment for the primary and secondary prevention of cardiovascular disease: summary of updated NICE guidance. BMJ. (2014) 349:g4356. doi: 10.1136/bmj.g4356 25035388

[21] Abdulaziz Qari F. Thyroid hormone profile in patients with acute coronary syndrome. Iran Red Crescent Med J. (2015) 17:e26919. doi: 10.5812/ircmj 26421178 PMC4584079

[22] Brozaitiene J Mickuviene N Podlipskyte A Burkauskas J Bunevicius R. Relationship and prognostic importance of thyroid hormone and N-terminal pro-B-Type natriuretic peptide for patients after acute coronary syndromes: a longitudinal observational study. BMC Cardiovasc Disord. (2016) 16:45. doi: 10.1186/s12872-016-0226-2 26892923 PMC4757967

[23] Cappola AR Desai AS Medici M Cooper LS Egan D Sopko G. Thyroid and cardiovascular disease: Research agenda for enhancing knowledge, prevention, and treatment. Circulation. (2019) 139:2892–909. doi: 10.1161/CIRCULATIONAHA.118.036859 PMC6851449 31081673

[24] Cappola AR Arnold AM Wulczyn K Carlson M Robbins J Psaty BM. Thyroid function in the euthyroid range and adverse outcomes in older adults. J Clin Endocrinol Metab. (2015) 100:1088–96. doi: 10.1210/jc.2014-3586 PMC4333030 25514105

[25] Merke A Jürgen M Guenther S Winfried M. Free thyroid hormones and mortality in caucasians undergoing angiography: the ludwigshafen risk and cardiovascular health (luric) study. Endocr Pract. (2017) 23:288–98. doi: 10.4158/EP161217.OR 27849383

[26] Pasqualetti G Calsolaro V Bernardini S Linsalata G Bigazzi R Caraccio N. Degree of peripheral thyroxin deiodination, frailty, and long-term survival in hospitalized older patients. J Clin Endocrinol Metab. (2018) 103:1867–76. doi: 10.1210/jc.2017-02149 29546287

[27] Nie X Ma X Xu Y Shen Y Wang Y Bao Y. Increased serum adipocyte fatty acid-binding protein levels are associated with decreased sensitivity to thyroid hormones in the euthyroid population. Thyroid. (2020) 30:1718–23. doi: 10.1089/thy.2020.0011 32394790

[28] Yu T Tian C Song J He D Wu J Wen Z. Value of the fT3/fT4 ratio and its combination with the GRACE risk score in predicting the prognosis in euthyroid patients with acute myocardial infarction undergoing percutaneous coronary intervention: a prospective cohort study. BMC Cardiovasc Disord. (2018) 18:181. doi: 10.1186/s12872-018-0916-z 30200880 PMC6131820

[29] Geng H Zhang X Wang C Zhao M Yu C Zhang B. Even mildly elevated TSH is associated with an atherogenic lipid profile in postmenopausal women with subclinical hypothyroidism. Endocr Res. (2015) 40:1–7. doi: 10.3109/07435800.2013.879166 24679183

[30] Wang P Xu-Y Guan Y-F Zhao Y Li Z-Y Lan X-H. Vascular smooth muscle cell apoptosis is an early trigger for hypothyroid atherosclerosis. Cardiovasc Res. (2014) 102:448–59. doi: 10.1093/cvr/cvu056 24604622

[31] Ning Y Jia Y Yang Y Wen W Huang M Liu S. Thyroid hormones inhibit apoptosis of macrophage induced by oxidized low-density lipoprotein. Biofactors. (2022) 48:86–99. doi: 10.1002/biof.1803 34882872

[32] Kozdag G Ural D Vural A Agacdiken A Kahraman G Sahin T. Relation between free triiodothyronine/free thyroxine ratio, echocardiographic parameters and mortality in dilated cardiomyopathy. Eur J Heart Fail. (2005) 7:113–8. doi: 10.1016/j.ejheart.2004.04.016 15642542

[33] Amsterdam EA Wenger NK Brindis RG Casey DE Jr Ganiats TG Holmes DR Jr. 2014 AHA/ACC guideline for the management of patients with non-ST-elevation acute coronary syndromes: a report of the american college of cardiology/american heart association task force on practice guidelines. J Am Coll Cardiol. (2014) 64:e139–228. doi: 10.1016/j.jacc.2014.09.017 25260718

[34] Iervasi G Pingitore A Landi P Raciti M Ripoli A Scarlattini M. Low-T3 syndrome: a strong prognostic predictor of death in patients with heart disease. Circulation. (2003) 107:708–13. doi: 10.1161/01.CIR.0000048124.64204.3F 12578873

[35] Anderson JLC Gruppen EG van Tienhoven-Wind L de Vries MFEH Gansevoort RT. Glomerular filtration rate is associated with free triiodothyronine in euthyroid subjects: Comparison between various equations to estimate renal function and creatinine clearance. Eur J Intern Med. (2018) 48:94–9. doi: 10.1016/j.ejim.2017.10.009 29079274

[36] Dimitriadis G Baker B Marsh H Mandarino L Rizza R Bergman R. Effect of thyroid hormone excess on action, secretion, and metabolism of insulin in humans. Am J Physiol. (1985) 248:E593–601. doi: 10.1152/ajpendo.1985.248.5.E593 3887944

[37] O'Meara NM Blackman JD Sturis J Polonsky KS. Alterations in the kinetics of C-peptide and insulin secretion in hyperthyroidism. J Clin Endocrinol Metab. (1993) 76:79–84. doi: 10.1210/jc.76.1.79 8421108

[38] Levin RJ Smyth DH. The effect of the thyroid gland on intestinal absorption of hexoses. J Physiol. (1963) 169:755–69. doi: 10.1113/jphysiol.1963.sp007294 PMC1368798 14103558

[39] Matty AJ Seshadri B. Effect of thyroxine on the isolated rat intestine. Gut. (1965) 6:200–2. doi: 10.1136/gut.6.2.200 PMC1552256 14279726

[40] Mokuno T Uchimura K Hayashi R Hayakawa N Makino M Nagata M. Glucose transporter 2 concentrations in hyper- and hypothyroid rat livers. J Endocrinol. (1999) 160:285–9. doi: 10.1677/joe.0.1600285 9924198

[41] LeFevre ML. Screening for thyroid dysfunction: U.S. Preventive Services Task Force recommendation statement. Ann Intern Med. (2015) 162:641–50. doi: 10.7326/M15-0483 25798805

