# Supplementary material for: Free triiodothyronine to free thyroxine ratio as a marker of poor prognosis in euthyroid patients with acute coronary syndrome and diabetes after percutaneous coronary intervention
Source: Front Endocrinol (Lausanne). 2024 Apr 8;15:1322969. doi: 10.3389/fendo.2024.1322969 (PMC11036861; doi:10.3389/fendo.2024.1322969)
Supplement: Supplementary Table 1 — Baseline percutaneous coronary intervention characteristics of the study population grouped by FT3/FT4 ratio. [file Table_1.doc]

|  | Q1(n=595)  (fT3/fT4≥0.45) | Q2(n=594)  (0.39≤fT3/fT4<0.45) | Q3(n=597)  (fT3/fT4 <0.39) | *P* |
| --- | --- | --- | --- | --- |
| Diseased vessels number | 2.29±0.89 | 2.21±0.91 | 2.21±0.87 | 0.648 |
| Stent number | 1.65±0.93 | 1.62±0.92 | 1.58±0.88 | 0.518 |
| Stent length | 39.45±25.93 | 38.67±26.51 | 37.79±24.56 | 0.341 |
| Mean stent diameter | 2.9±0.42 | 2.92±0.41 | 2.95±0.43 | 0.201 |
| Minimum stent diameter | 2.79±0.44 | 2.81±0.44 | 2.84±0.44 | 0.139 |
| LM (n, %) | 46(7.7) | 40(6.7) | 43(7.2) | 0.106 |
| LAD (n, %) | 478(80.1) | 469(79.0) | 467(78.5) | 0.356 |
| LCX (n, %) | 385(64.5) | 362(60.9) | 338(56.8) | 0.233 |
| RCA (n, %) | 415(69.5) | 404(68.0) | 413(69.4) | 0.664 |
| Graft (n, %) | 5(0.8) | 9(1.5) | 6(1.0) | 0.077 |
| Bifurcation disease (n, %) | 65(10.9) | 73(12.3) | 63(10.6) | 0.216 |
| Chronic total occlusion (n, %) | 69(11.6) | 80(13.5) | 76(12.8) | 0.393 |
| Multivessel lesion (n, %) | 468(78.4) | 435(73.2) | 454(76.3) | 0.101 |

Continuous variables were represented as mean ± standard deviation; Categorical variables were represented by percentiles. LM: Left main Coronary Artery; LAD: Left Anterior Descending Artery; LCX: Left Circumflex Artery.
